# Characterization of a eukaryotic translation initiation factor 5A homolog from *Tamarix androssowii* involved in plant abiotic stress tolerance

**DOI:** 10.1186/1471-2229-12-118

**Published:** 2012-07-26

**Authors:** Liuqiang Wang, Chenxi Xu, Chao Wang, Yucheng Wang

**Affiliations:** 1 State Key Laboratory of Tree Genetics and Breeding (Northeast Forestry University), 26 Hexing Road, Harbin, China

## Abstract

**Background:**

The eukaryotic translation initiation factor 5A (eIF5A) promotes formation of the first peptide bond at the onset of protein synthesis. However, the function of eIF5A in plants is not well understood.

**Results:**

In this study, we characterized the function of *eIF5A* (*TaeIF5A1*) from *Tamarix androssowii*. The promoter of *TaeIF5A1* with 1,486 bp in length was isolated, and the *cis*-elements in the promoter were identified. A WRKY (TaWRKY) and RAV (TaRAV) protein can specifically bind to a W-box motif in the promoter of *TaeIF5A1* and activate the expression of *TaeIF5A1*. Furthermore, *TaeIF5A1*, *TaWRKY* and *TaRAV* share very similar expression pattern and are all stress-responsive gene that functions in the abscisic acid (ABA) signaling pathway, indicating that they are components of a single regulatory pathway. Transgenic yeast and poplar expressing *TaeIF5A1* showed elevated protein levels combined with improved abiotic stresses tolerance. Furthermore, *TaeIF5A1*-transformed plants exhibited enhanced superoxide dismutase (SOD) and peroxidase (POD) activities, lower electrolyte leakage and higher chlorophyll content under salt stress.

**Conclusions:**

These results suggested that *TaeIF5A1* is involved in abiotic stress tolerance, and is likely regulated by transcription factors TaWRKY and TaRAV both of which can bind to the W-box motif. In addition, *TaeIF5A1* may mediate stress tolerance by increasing protein synthesis, enhancing ROS scavenging by improving SOD and POD activities, and preventing chlorophyll loss and membrane damage. Therefore, eIF5A may play an important role in plant adaptation to changing environmental conditions.

## Background

Eukaryotic initiation factor 5A (eIF5A) is a small protein ubiquitously present throughout the eukaryotic kingdom. The protein was initially identified in rabbit reticulocytes as a factor involved in formation of the first peptide bond [1,2]. EIF5A is a highly conserved protein and contains the post-translationally synthesized amino acid hypusine [3]. Molecular and biochemical studies in yeast and mammalian cells demonstrated that eIF5A is synthesized as an inactive precursor that is activated by a post-translational hypusine modification that is only detected in the eIF5A protein, and consists of a two-step sequential reaction catalyzed by deoxyhypusine synthase (DHS, EC:2.5.1.46) and deoxyhypusine hydroxylase (DHH, EC1.14.99.29) [4,5].

The precise cellular function of eIF5A is not fully understood. It was originally considered to be a translation initiation factor as it can stimulate methionyl-puromycin synthesis *in vitro* and transiently attach to ribosomes to begin eukaryotic cellular protein synthesis. In addition, eIF5A promotes the formation of the first peptide bond at the initiation of protein synthesis [1]. Recent studies have demonstrated that eIF5A dysfunction significantly decreases protein synthesis in yeast, and that eIF5A promotes translation elongation in *Saccharomyces cerevisiae*[6-8]. Henderson and Hershey found that although eIF5A is not required for protein synthesis, eIF5A can stimulate the process by about 2- to 3-fold. They further draw a conclusion that the polysome profiles observe during and after eIF5A depletion are diagnostic for a role in initiation [9]. In addition, eIF5A is involved in cellular proliferation and apoptosis [10], promotes cell viability and cell growth [11] and the synthesis of proteins involved in progression of the cell cycle [12]. Moreover, eIF5A proteins are found to facilitate protein synthesis by participating in the nuclear export of specific mRNAs [5]. Furthermore, eIF5A proteins also play a role in RNA binding, and contain a C-terminal domain with a structure that resembles an oligonucleotide-binding fold [13].

Plant eIF5A proteins are also highly conserved that are involved in multiple biological processes, including protein synthesis regulation, translation elongation, mRNA turnover and decay, cell proliferation, leaf and root growth, seed yield, leaf, flower and fruit senescence and programmed cell death [14-16]. Ma *et al*. showed that eIF5A plays roles in supporting plant growth and in regulating responses to sub-lethal osmotic and nutrient stress [17]. Valentini *et al.* showed that eIF5A is involved in the WSC/PKC1 signaling pathway that controls cell wall integrity or related processes, and plays a role in cell wall formation [18]. Hopkins *et al.* reported that eIF5A plays a vital role in signal transduction pathways involved in pathogen-induced cell death and in the development of plant disease symptoms. Plant *eIF5A* genes are also involved in abiotic stress responses [3]. For instance, Xu *et al.* showed that transgenic *Arabidopsis* plants overexpressing *RceIF5A* show improved resistance to heat, oxidative and osmotic stresses, while the plants with reduced *eIF5A* expression (three *AteIF5A* isoforms in *Arabidopsis* are down-regulated) are more susceptible to these stresses [16]. Chou *et al.* reported that salt and heavy metal stresses induce the expression of rice *eIF5A* genes, *OseIF5A-1* and *OseIF5A-2*, suggesting that they are involved in stress tolerance [19].

However, little is known of the upstream regulators or its regulatory network, and its role in stress tolerance. In addition, if eIF5A does in fact confer stress tolerance in plants, the physiological changes mediated by eIF5A deserve further study.

*Tamarix* (Tamaricaceae) species, which include small trees or shrubs, are widely distributed in the saline soils of drought-stricken areas of Central Asia and China. *Tamarix androssowii* Litvinov is highly tolerant to abiotic stresses, such as salinity, drought and high temperatures. These characteristics make the species a suitable source of stress tolerance genes and for investigating endogenous stress resistance mechanisms.

In the present study, we cloned and functionally characterized an *eIF5A* from *T. androssowii*. We showed that *TaeIF5A1* is a stress-responsive gene involved in the ABA signal transduction pathway. *TaWRKY* and *TaRAV* can active the expression of *TaeIF5A1*. In addition, *TaeIF5A1* facilitates protein synthesis and regulates several physiological pathways to improve stress tolerance. This study reveals a physiological role for eIF5A and defines a possible mechanism for eIF5A-mediated stress tolerance in plants.

## Results

### Cloning and analysis of *TaeIF5A1* and its promoter

**Figure 1:**
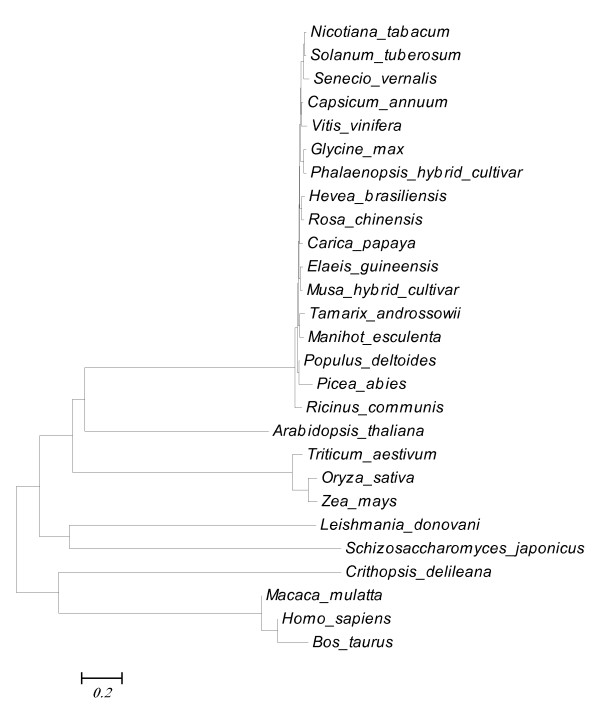
**Phylogenetic tree of TaeIF5A1 and other eIF5As from different species.** All protein sequences used in the phylogenetic analysis were retrieved from GenBank, and their GenBank accession numbers are as follows: *Tamarix androssowii* (AAT01416), *Ricinus communis* (XP_002514712), *Populus deltoides* (ACM79935), *Picea abies* (AAX92694), *Manihot esculenta* (AAK55848), *Glycine max* (ACJ76773), *Hevea brasiliensis* (AAQ08194), *Rosa chinensis* (ABM53472), *Oryza sativa* (CAB96075), *Nicotiana tabacum* (CAA45105), *Senecio vernalis* (CAB65463), *Capsicum annuum* (AAS48586), *Carica papaya* (ABS01354), *Elaeis guineensis* (ACF06454), *Solanum tuberosum* (ABB16995), *Musa hybrid cultivar* (ACP31200), *Phalaenopsis hybrid cultivar* (CAL69910), *Zea mays* (NP_001105606), *Arabidopsis thaliana* (AAG53646), *Triticum aestivum* (AAZ95172), *Vitis vinifera* (XP_002273265), *Crithopsis delileana* (ABB90163), *Homo sapiens* (NP_112594), *Leishmania donovani* (ADJ39999), *Schizosaccharomyces japonicus* (XP_002173495), *Macaca mulatta* (AFH28009), *Bos taurus* (NP_001069354).

The *TaeIF5A1* gene (GenBank number: AY587771), 801 bp in length and encoding a 159 aa protein with a predicted molecular weight of 17.33 kDa, was cloned from a *T. androssowii*. To investigate the homology of known eIF5A proteins, a phylogenetic tree was constructed (Figure 1), which showed that TaeIF5A1 is most similar to the eIF5A from *Manihot esculenta* and has a long evolutional distance from eIF5As of yeast and mammalian. However, there is little published information about the biological functions of these eIF5A proteins and their molecular functions await further study.

The promoter of *TaeIF5A1* was cloned using TAIL-PCR, and a promoter fragment with 1486 bp (from −1 to −1486) in length was obtained. We identified diverse *cis*-elements in the promoter, including ARR1AT, DOFCOREZM, MYB1AT, MYBCORE and W-box (Additional file 1) using PLACE ( http://www.dna.affrc.go.jp) [20]. The W-box sequence “CTGACT” was identified in *TaeIF5A1* promoter that shows high binding affinity to WRKY [21].

### Expression of the *TaeIF5A1* gene

**Figure 2:**
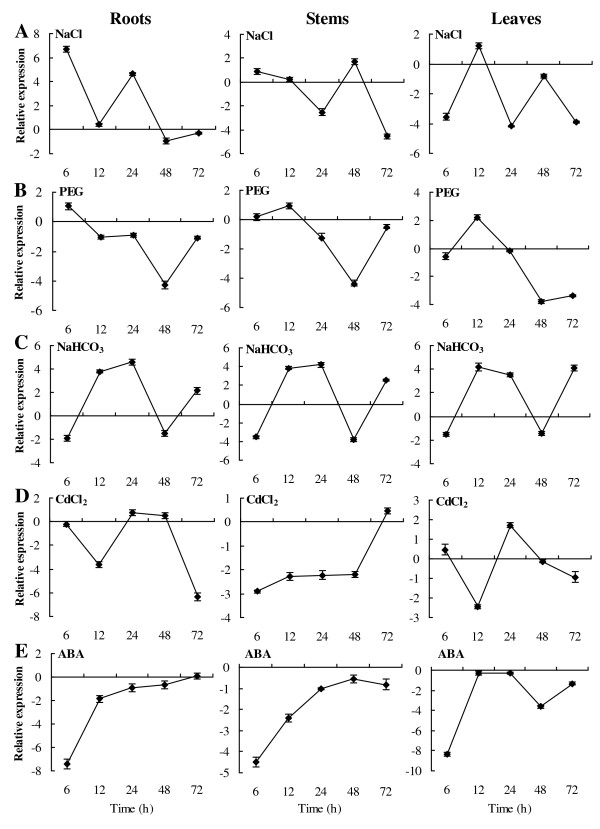
**Analysis of TaeIF5A1 expression in response to different abiotic stresses.A–E.** Two-month-old seedlings were watered with one of the following solutions containing 0.4 M NaCl, 20% PEG6000, 0.3 M NaHCO_3_, 150 μM CdCl_2_ , or 150 μM ABA for the indicated times. The relative expression level = log2 (transcription level under stress treatment / transcription level under control conditions). The error bars were obtained from multiple replicates of the real-time PCR.

Real-time RT-PCR showed that *TaeIF5A1* can be detected in roots, stem and leaves, and is differentially regulated by different abiotic stresses. The expression of *TaeIF5A1* was induced in roots by salt stress at 6 or 24 h, but not at other time points. In stems, *TaeIF5A1* was down-regulated after 24 and 72 h of NaCl stress. Moreover, *TaeIF5A1* was strongly down-regulated in leaves after 6, 24 and 72 h of NaCl stress, but its expression was unaffected at other time points (Figure 2A). The *TaeIF5A1* was down-regulated in roots, stems and leaves following exposure to PEG stress for 24–72 h (Figure 2B). Interestingly, *TaeIF5A1* exhibited the same expression pattern in roots, stems, and leaves under NaHCO_3_ stress, being up-regulated after 12, 24 and 72 h of stress and down-regulated at all other time points (Figure 2C). Following CdCl_2_ stress, *TaeIF5A1* was generally down-regulated in roots, stems and leaves (Figure 2D). ABA treatment induced a marked inhibition of *TaeIF5A1* expression in roots, stems and leaves by 6 h, followed by recovery thereafter (Figure 2E).

**Figure 3:**
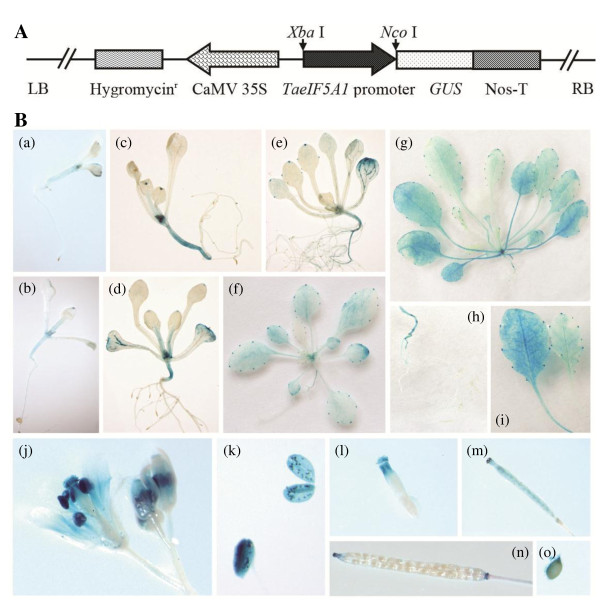
**Analysis of the promoter activity of TaeIF5A1 in transgenic Arabidopsis plants expressing the TaeIF5A1 promoter::GUS construct.A.** Schematic map of the T-DNA inserted in the pCAMBIA1301 binary vector, which was used for *Arabidopsis* transformation. **B.** The spatial expression of *TaeIF5A1* at different growth stages and in different organs or tissues of transgenic *Arabidopsis* plants expressing the *TaeIF5A1 promoter::GUS* construct. (a) Three-day-old seedling; (b) One-week-old seedling; (c) Ten-day-old seedling; (d) Two-week-old seedling; (e) Three-week-old seedling; (f) Four-week-old seedling; (g) Five-week-old seedling; (h) Roots from a 5-week-old plant; (i) Two rosette leaves from a 5-week-old plant; (j) The whole flower cluster; (k) Anther in pollen grains; (l) Pistil; (m) The intact pistil; (n) The intact silique; (o) The seed of Arabidopsis.

To further investigate the promoter activity of *TaeIF5A1*, the transgenic *Arabidopsis* plants expressing *GUS* under the control of *TaeIF5A1* promoter were analyzed using GUS staining (Figure 3B). In young *Arabidopsis* (less than 3-week old), GUS activity was mainly confined to the cotyledons, the main root, the leaf tips, the tips of leaf teeth, veins and hydathodes (Figure 3Ba–e). GUS activity was present throughout the whole plant in five-week-old plants, and showed a step-wise reduction in both expression area and level in three-week-old and four-week-old plants (Figure 3Be–g). Further, changes in GUS expression pattern were observed during the development of the reproductive organs (Figure 3Bj–o). In flowers, high GUS expression was predominantly observed in the pistils, stigma, stamens, anther and petals, but not in the sepals (Figure 3Bj–l). In the siliques, GUS activity was present in the adhesion zones (Figure 3Bn). Consistent with real-time PCR results (Figure 2), these results indicated that *TaeIF5A1* is expressed in all the tissues including leaves, roots, stems at all growth stages and reproductive organs.

### Targeting TaeIF5A1 to nucleus and cytoplasm

**Figure 4:**
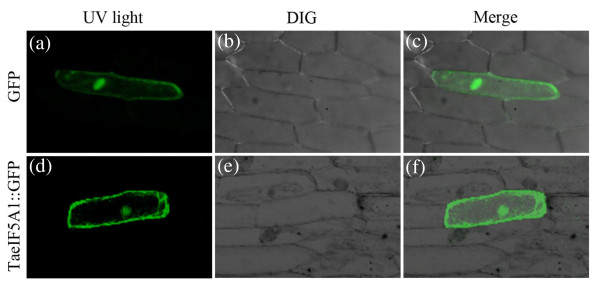
**Subcellular localization of TaeIF5A1.** The TaeIF5A1–GFP fusion construct and the GFP control plasmid were introduced into the onion epidermal cells by particle bombardment. Expression of the introduced genes was examined after 48 h by fluorescence and light microscopy. **(a, d)** GFP fluorescence; **(b, e)** onion peel cells imaged under bright field; **(c, f)** merge of bright field and fluorescence.

The subcellular localization of TaeIF5A1 was determined using the *TaeIF5A1*::*GFP* fusion gene under the control of the CaMV 35S promoter. The *TaeIF5A1*::*GFP* fusion gene and *GFP* control were transformed into onion epidermal cells by particle bombardment. We detected the green fluorescent signal of TaeIF5A1–GFP in the nucleus and uniformly distributed throughout transformed cells (Figure 4), suggesting that the TaeIF5A1 protein showed nuclear and cytoplasm localization.

### Analysis of the upstream regulator of *TaeIF5A1*

**Figure 5:**
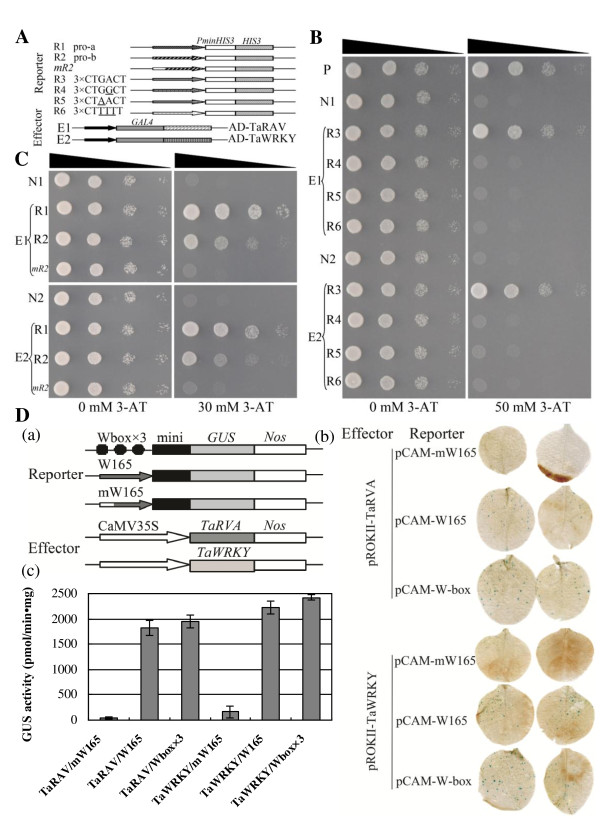
**Yeast one-hybrid analyses of the factors binding to W-box elements.A.** Scheme of reporter and effector vectors. Fragments of the *TaeIF5A1* promoter were inserted into the upstream of the *His3* reporter gene. Promoter fragments of 461 bp and 165 bp containing the W-box motif (construct R1, R2) and promoter fragment of 165 bp containing the mutated sequence (construct *mR2*) were tested. The W-box and mutated W-boxes were respectively cloned into the upstream of the *His3* reporter gene: W-box (construct R3); the mutants (constructs R4–R6). The effector vectors, E1: pGADT7-Rec2 harboring *TaRAV*; E2: pGADT7-Rec2 harboring *TaWRKY*. **B.** TaRAV and TaWRKY interaction with W-box (R3) or mutants (R4–R6) sequences. **C.** Determination of TaRAV and TaWRKY binding to the promoter fragments containing W-box or mutated motif (R1, R2, *mR2*). The effector and the reporter constructs were co-transformed into yeast strain Y187. Positive transformants were determined by spotting serial dilutions (1:1, 1:10, 1:100, 1:1000) of yeast onto SD/-His/-Leu/-Trp plates with 3-AT. Negative controls: N1, p53HIS2 + E1 (AD-TaRAV); N2, p53HIS2 + E2 (AD-TaWRKY); Positive control: P, p53HIS2 + pGAD-Rec2-53. **D.** TaRAV and TaWRKY binding to the W-box and the promoter fragment containing the W-box motif in tobacco leaves. (a) Construction of reporter and effector plasmids for transient trans-activation assays; (b) GUS staining of tobacco leaves co-transformed with reporter and effector plasmids; (c) GUS activity assay of the co-expression of effector and reporter plasmids. The data represent mean values of three independent experiments.

A W-box element was found in the *TaeIF5A1* promoter, suggesting that *TaeIF5A1* may be regulated by transcription factors that interact with W-box motif. To investigate the upstream regulator of *TaeIF5A1*, yeast one-hybrid assay was performed using the pHIS2-*cis* (containing triple tandem repeats of the W-box) reporter vector as bait to screen a *Tamarix* cDNA library. In total, two genes were found to specifically bind the W-box motif (Figure 5B): WRKY transcription factor (TaWRKY, GenBank number: JQ040808) and AP2/ERF and B3 domain-containing transcription factor (TaRAV, GenBank number: JQ040809).

To characterize the specific interaction between the W-box and TaWRKY and TaRAV, we mutated the core W-box motif “TGAC” to “TGGC”, “TAAC” and “TTTT”. Both TaWRKY and TaRAV could bind the W-box motif, but they failed to bind to each of the mutants (Figure 5B), indicating that both “G” and “A” in “TGAC” are necessary for W-box recognition. These results indicate that TaWRKY and TaRAV can specifically bind to the W-box.

To further investigate whether these two genes can activate the expression of *TaeIF5A1*, promoter fragments of 461 and 165 bp in length containing the W-box motif, and promoter fragment of 165 bp in length containing mutated core sequence “TTTT” were respectively inserted into pHIS2, and the interactions between these promoter fragments and the two genes were determined using the yeast one-hybrid system. We found that both TaWRKY and TaRAV can specifically bind to the two promoter fragments containing the W-box, but failed in binding to the promoter fragment containing the mutated core sequence “TTTT” and the control (N1, N2) (Figure 5C), indicating that they may regulate the expression of *TaeIF5A1* through binding to the W-box motif in the promoter of *TaeIF5A1*.

To further confirm the above interactions, we co-transformed the effector constructs (pROKII-TaRVA or pROKII-TaWRKY) in which *TaRVA* or *TaWRKY* is driven by 35S promoter and their corresponding reporter plasmids (pCAM-W-box, pCAM-W165, pCAM-mW165) into tobacco leaves. Histochemical staining and GUS activity assay showed that the *GUS* gene was activated in tobacco cells when co-transformation of pROKII-TaRVA or pROKII-TaWRKY with pCAM-W-box and pCAM-W165; however co-transformation of pROKII-TaRVA or pROKII-TaWRKY with pCAM-mW165 failed in GUS activation (Figure 5D). These data clearly indicated that both TaRVA and TaWRKY can activate expression of *TaeIF5A1* by binding to W-box motif in its promoter.

**Figure 6:**
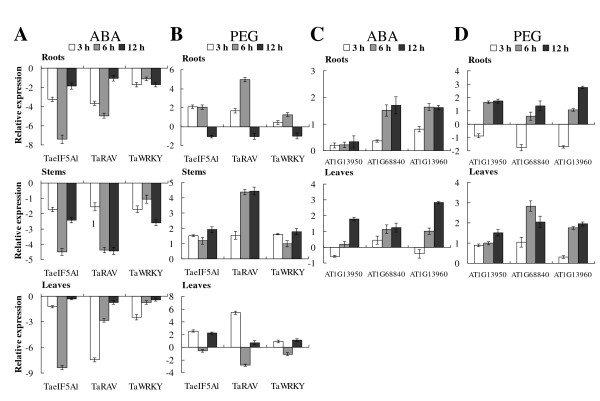
**Expression patterns of TaeIF5A1 and its upstream regulator genes (TaRAV and TaWRKY) and their Arabidopsis homologs.A–B.** Expression patterns of *TaeIF5A1* and its upstream regulator genes (*TaRAV* and *TaWRKY*) in the roots, stems and leaves of 2-month-old *T*. *hispida* seedlings subjected to 150 μM ABA or 20% PEG6000 treatment. **C–D.** Expression patterns of the AT1G13950, AT1G68840 and AT1G13960 genes in the roots and leaves of three week-old *Arabidopsis* seedlings subjected to 100 μM ABA or 20% PEG6000 treatment. AT1G13950, AT1G68840 and AT1G13960 in *Arabidopsis* are homolog to *TaeIF5A1*, *TaRAV* and *TaWRKY*, respectively. The relative expression level = log2 (transcription level under stress treatment / transcription level under control conditions). The error bars were obtained from multiple replicates of the real-time PCR.

The expression patterns of *TaRAV*, *TaWRKY* and *TaeIF5A1* were investigated using real-time RT-PCR. We found that both the expression of *TaRAV* and *TaWRKY* are induced by osmotic stress and negatively regulated by ABA treatment, suggesting that *TaRAV* and *TaWRKY* are stress response genes and involved in ABA signaling pathway. Moreover, *TaRAV*, *TaWRKY* and *TaeIF5A1* all share very similar expression patterns under different stress conditions (Figure 6A, B).

Given the facts that TaRAV and TaWRKY can activate the expression of *TaeIF5A1*, we next studied if this mechanism of transcriptional regulation is also maintained in the model plant *Arabidopsis*. Using BLASTX program in Tair, we identified the homologs of *TaeIF5A1*, *TaRAV* and *TaWRKY* in *Arabidopsis* are AT1G13950, AT1G68840 and AT1G13960, respectively. Real time RT-PCR showed that as in *T*. *androssowii*, AT1G13950, AT1G68840 and AT1G13960 also shared a very similar expression patterns in *Arabidopsis* when exposed to ABA and osmotic stress (Figure 6C, D).

### Functional analysis of the *TaeIF5A1* gene using a *S. cerevisiae* expression system

**Figure 7:**
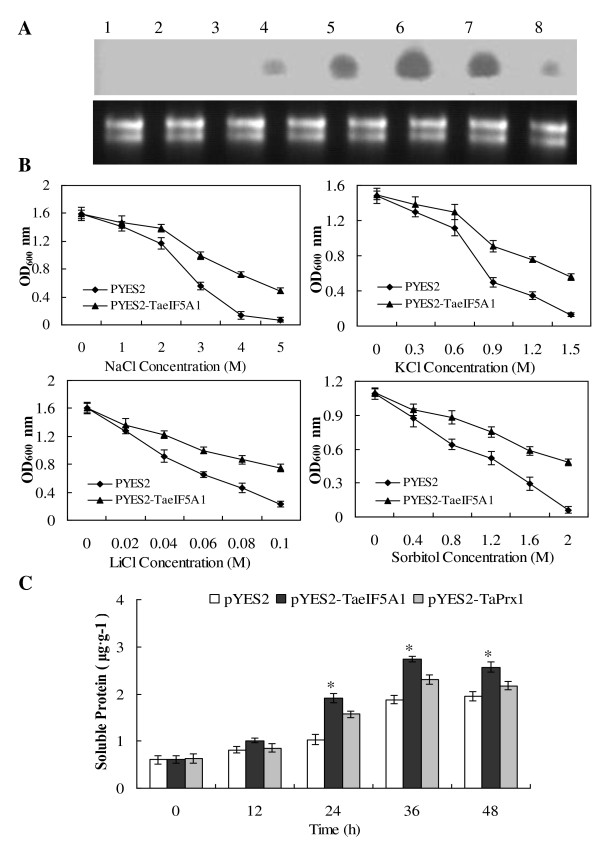
**Functional characterization of TaeIF5A1 using the S. cerevisiae expression system.A.** RNA gel blot analysis of the expression of exogenous *TaeIF5A1* in yeast at different induction times. 1–2, empty pYES2 transformants were cultured in induction medium (SC-Ura-/Gal 2%) or suppression medium (SC-Ura-/Glu 2%) at 30°C for 36 h. 3–8, yeast transformants harboring *TaeIF5A1* were cultured in induction medium at 30°C for 0, 12, 24, 36, 48 and 60 h. **B.** Abiotic stress tolerance analyses of *TaeIF5A1*. Comparison of growth between two transgenic yeast lines (harboring empty pYES2 or pYES2-TaeIF5A1). Growth was detected by measuring the absorbance at 600 nm after culturing at 30°C for 24 h in liquid medium supplemented with different concentrations of NaCl, KCl, LiCl or sorbitol. **C.** Investigation of the effect of TaeIF5A1 on protein synthesis. Yeast transformants harboring *TaeIF5A1*, *TaPrx1* (positive control), or empty pYES2 were cultured in induction medium at 30°C for 0, 12, 24, 36 and 48 h for soluble protein content analysis. * means significant difference (P < 0.05) between yeast transformants harboring *TaeIF5A1* and *TaPrx1*.

Yeast transformants harboring *TaeIF5A1* or the empty pYES2 vector were generated to investigate the role of *TaeIF5A1* in stress tolerance*.* RNA gel blot showed that *TaeIF5A1* can be induced in yeast cells, and reached a peak of expression after induction for 36 h (Figure 7A). Therefore, 36 h was selected as a suitable induction time. Yeast transformants harboring *TaeIF5A1* or empty vector were grown on induction medium for 36 h, and then treated with different stress-inducing agents. Yeast cells expressing *TaeIF5A1* exhibited better growth than control did under NaCl, KCl, LiCl and sorbitol stress conditions (Figure 7B), suggesting that expression of *TaeIF5A1* in yeast increases tolerance to abiotic stresses.

We next compared the soluble protein content in three yeast transformants harboring *TaeIF5A1*, *TaPrx1* (as control) or empty pYES2 (as control for investigating if the exogenous gene expressed). The protein content of yeast transformants harboring *TaeIF5A1* was the highest, followed by transformants harboring *TaPrx1* then empty vector (Figure 7C). Transformants harboring empty pYES2 failed to produce an exogenous gene and exhibited the lowest overall protein expression; the two other transformed strains expressed greater levels of protein, suggesting that the exogenous genes had been expressed. Yeast transformants harboring *TaeIF5A1* displayed significantly (P < 0.05) higher overall protein levels than those harboring *TaPrx1*, indicating that the *TaeIF5A1* expression increases protein level in yeast cells.

### Overexpression of *TaeIF5A1* improves salt tolerance in transgenic plants

**Figure 8:**
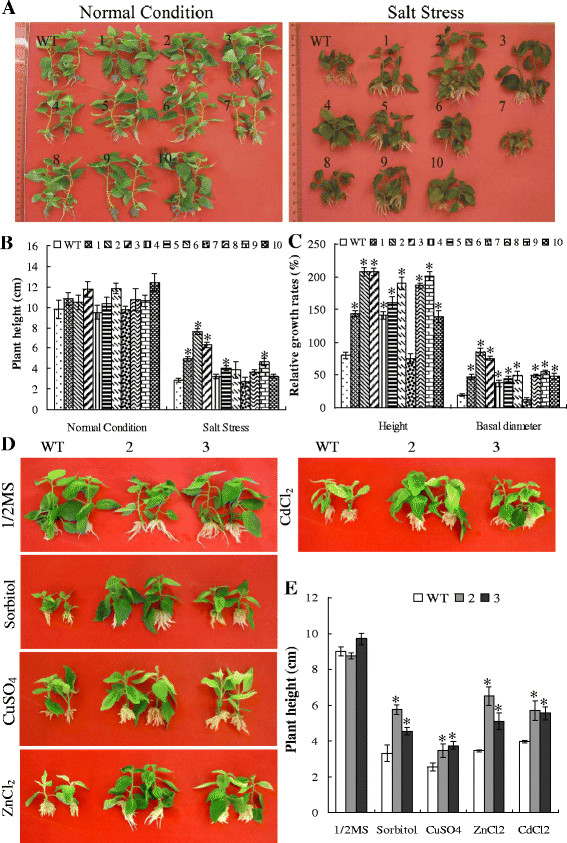
**Effects of the overexpression of TaeIF5A1on abiotic stress tolerance in transgenic poplar plants.A–B.** The seedlings of WT and transgenic lines grown on 1/2MS medium supplemented with 0 and 0.6 % NaCl for 20 d, the phenotypes of plantlets were photographed and the height was measured. **C.** Comparison of the relative rates of growth in height and basal diameter in WT and transgenic poplar lines. The plantlets with similar height from each line and control plants (sample size of 10 plantlets) growing in soil were treated with 0.8% NaCl solution for 30 d then watered normally, the height and basal diameter were measured after 90 d of treatment. RGH or RDG were calculated as: (final value - baseline value) / baseline value × 100 and presented as a percentage. **D–E.** The plantlets of WT and transgenic plants (2 and 3) with similar size were grown on 1/2MS medium supplied with 300 μM of CuSO_4_, CdCl_2_, 1 mM of ZnCl_2_, and 200 mM of sorbitol. After 16 d of stress, the phenotypes of plantlets were photographed and the height was measured. The error bars represent standard deviations of the mean measurements. * means significant difference (P < 0.05) between transgenic lines and WT plants. WT, wild type poplar plants; 1–10, ten lines of transgenic poplar plants.

To investigate whether overexpression of *TaeIF5A1* in plants enhances stress tolerance, transgenic poplar plants expressing the *TaeIF5A1* were generated. DNA and RNA gel blot analyses were conducted to confirm the integration and expression of exogenous *TaeIF5A1* in transgenic plants (Additional file 2B, C). Salt tolerance test on tube seedlings showed that there was no difference in height growth under normal growth conditions. However, under salt stress conditions, many transgenic lines exhibited significantly increased height growth relative to WT plants (Figure 8A, B). In addition, salt tolerance test on plantlets in soil indicated that all the transgenic lines except line 7 displayed significantly improved height and basal diameter growth than control did (Figure 8C). All these results suggested that salt tolerance of the transgenic lines was improved due to the overexpression of *TaeIF5A1*.

### Additional abiotic stress tolerance assays

Transgenic and WT plantlets were treated with CuSO_4_, CdCl_2_ and ZnCl_2_ or sorbitol for 16 d, and then their growth was compared. We found no difference in growth height between WT and transgenic plants under normal growing condition. However, relative to the WT, the transgenic plants exhibited greatly increased height growth under the various stress conditions (Figure 8D, E), indicating that plants overexpressing *TaeIF5A1* possess increased tolerance to these forms of abiotic stresses.

### Measurement of soluble protein content

**Figure 9:**
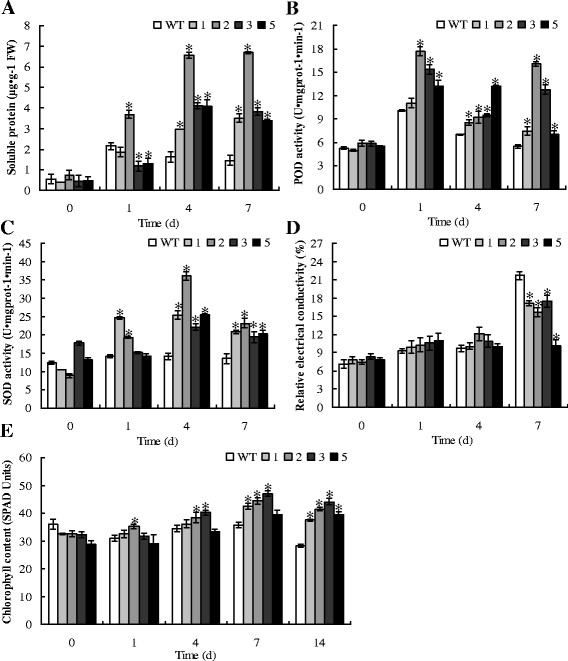
**Physiological analyses of WT plants and four transgenic *TaeIF5A1 *poplar lines.A–E.** Comparison of soluble protein levels, POD activity, SOD activity, electrolyte leakage and relative chlorophyll content (RCC) between WT and transgenic poplar plants. Plantlets (60–100 cm in height, each sample contains at least ten plantlets) from wild-type (WT) and transgenic plants (1, 2, 3 and 5) grown in soil were watered with 0.8% (w/v) NaCl for 0, 1, 4, 7 and 14 d, respectively. The error bars represent standard deviations of the mean measurements. * means significant difference (P < 0.05) between WT and transgenic lines plants.

Prior to salt stress, soluble protein levels in transgenic plants did not significantly differ from those of WT plants. However, after 4 d and 7 d of salt stress, all transgenic lines contained significantly (P < 0.05) higher levels of soluble protein than WT plants (Figure 9A), indicating that overexpression of *TaeIF5A1* greatly increases soluble protein levels in transgenic plants under salt stress conditions compared to WT plants.

### POD activity assay

Transgenic and WT plants had similar POD activity prior to salt stress. Under salt stress conditions, POD activity in WT plants was transiently elevated after 1 d of stress, and subsequently recovered. However, in all transgenic plants POD activity was markedly improved and was significantly (P < 0.05) higher than that in the WT during the entire 4–7 d period of exposure to stress conditions (Figure 9B).

### SOD activity comparison

SOD activity in the transgenic lines was either higher or lower than that of WT plants prior to salt stress (Figure 9C). Under salt stress conditions, SOD activity in WT plants did not change; however SOD activity was increased in transgenic lines, being significantly (P < 0.05) higher after 4 and 7 d of exposure to salt stress (Figure 9C), indicating that *TaeIF5A1* overexpression specifically increases SOD activity under salt stress condition.

### Electrolyte leakage assay

Electrolyte leakage was not significantly different (P > 0.05) between transgenic and WT plants prior to stress. Electrolyte leakage increased in both transgenic and WT plants under stress conditions, with maximal electrolyte leakage levels after 7 d of salt stress. However, the increase of electrolyte leakage in transgenic lines was significantly lower than in WT plants (Figure 9D). These results indicated that transgenic plants suffer less membrane damage under salt stress conditions compared to WT plants.

### Relative chlorophyll content (RCC) comparison

The RCC of WT plants did not notably vary following exposure to salt stress for 1–7 d, but markedly decreased after 14 d of exposure to stress. However, the RCC in transgenic plants increased over 4–7 d, and reached significantly (P < 0.05) higher levels than that of WT control plants under stress for 7–14 d (Figure 9E), indicating that *TaeIF5A1* overexpression may enhance chlorophyll levels and prevent chlorophyll loss under salt stress condition.

## Discussion

### *TaeIF5A1* is expressed in all tissues of plants, and tolerant to different abiotic stresses

In the present study, we found that the *TaeIF5A1* gene was significantly differentially regulated by NaCl, NaHCO_3_, PEG and CdCl_2_ treatments (Figure 2A–D), suggesting roles in the abiotic stress response. In addition, the expression of *TaeIF5A1* and its upstream regulators, *TaRAV* and *TaWRKY*, were strongly inhibited by ABA (Figure 6A), suggesting that they are involved in ABA-dependent signaling pathway. Moreover, both yeast transformants and transgenic plants expressing *TaeIF5A1* displayed increased tolerance to NaCl, KCl, ZnCl_2_, CuSO_4,_ CdCl_2_ and sorbitol stresses. Furthermore, *TaeIF5A1* gene is expressed in all the tissues including leaves, roots and stem at different growth stages, and also in reproductive organs (Figure 3B); therefore they may play role of stress tolerance in all of these tissues of plants.

### The W-box motif “CTGACT” can be specifically recognized by TaRAV and TaWRKY

*RAV* transcription factors contain an AP2/ERF domain in their N-terminal regions and a B3 domain in their C-termini. Some *RAV* genes can interact with the sequences “CAACA”, “CACCTG” and the GCC-box “AGCCGCC” [22], which are involved in plant defense pathways [23] and diverse bioprocesses such as flowering, germination and the early events of leaf senescence [24,25]. In the present study, a *RVA* gene, *TaRVA*, was found to bind to the W-box motif “CTGACT” (Figure 5v), and the binding is lost following “G” to “A” or “A” to “G” mutation of core “TGAC” motif (Figure 5B), suggesting a specific interaction.

*WRKY* genes play a variety of roles in plant developmental and physiological processes. Previous studies showed that most WRKY proteins can bind to the cognate *cis*-acting element “C/TTGACT/C” in the promoter or the 5’ untranslated regions of target genes [21]. Our results showed that a WRKY homolog (TaWRKY) can specifically bind to the W-box motif “CTGACT” and that the core “TGAC” motif is sufficient for binding (Figure 5B).

### The expression of *TaeIF5A1* is likely regulated by TaWRKY and TaRAV both of which can bind to the W-box

A W-box motif is present in the *TaeIF5A1* promoter, indicating that it may be regulated by W-box-binding transcription factors. Both yeast one-hybrid analysis and co-expression of reporter and effector genes demonstrated that two proteins, TaWRKY and TaRAV, which can specially bind to *TaeIF5A1* promoter fragments containing the W-box, but failed in binding to the same fragments containing the mutated core sequence “TTTT” (Figure 5C, D). These results clearly suggested that both TaWRKY and TaRAV can activate the expression of *TaeIF5A1* by binding to the W-box motif present in the promoter of *TaeIF5A1*. Moreover, the expression of *TaeIF5A1*, *TaWRKY* and *TaRAV* share very similar expression patterns, are all inhibited following ABA treatment and induced by osmotic stress (Figure 6A, B), suggesting that *TaeIF5A1*, *TaWRKY* and *TaRAV* may be components of a single regulatory pathway. Therefore, these combined results strongly suggest that *TaWRKY* and *TaRAV* are the upstream regulators of *TaeIF5A1* that can regulate the expression of *TaeIF5A1* through binding to the W-box motif in the *TaeIF5A1* promoter. In *Arabidopsis*, the homologs of *TaeIF5A1*, AT1G13950, also has three W-box motifs in its promoter (from −10 to −670), suggesting that it may also be regulated by *RAV* and *WRKY*. Interestingly, real time RT-PCR results showed that the AT1G13950, AT1G68840 and AT1G13960 all shared very similar expression profiles in response to ABA and osmotic stress (Figure 6C, D), implying that these genes may also be components of a single regulatory pathway. These results indicated that the *RAV* and *WRKY* activate the expression of *eIF5A* may be a conserved mechanism of transcriptional regulation, which is also maintained in the model plant *Arabidopsis*.

### *TaeIF5A1* facilitates protein synthesis to improve stress tolerance

In the present study, we introduced *TaeIF5A1* into yeast *S. cerevisiae* and poplar to investigate the role of *TaeIF5A1* in protein synthesis. We found that overall protein levels in *S. cerevisiae* and poplar expressing exogenous *TaeIF5A1* significantly improved compared with controls (Figure 7C and Figure 9A). These results suggest that *TaeIF5A1* facilitates protein synthesis. In plants, protein synthesis is highly sensitive to salt stress and overexpression of *eIF1A* can improve protein translation under stress conditions [1,26]. Increased salt tolerance is observed in plants transformed with *eIF1A*, indicating that protein synthesis positively correlates with stress tolerance [26]. In addition, protein accumulation provides a stored form of nitrogen that can be utilized to adjust osmotic potential [27], suggesting that a vital component of stress tolerance is the maintenance and enhancement of protein synthesis under stress conditions. Therefore, soluble protein levels may be closely associated with plant abiotic stress tolerance. These facts suggested that overexpression of *TaeIF5A1* improves both protein levels and stress tolerance supports this hypothesis.

### *TaeIF5A1* regulates some physiological pathways to improve stress tolerance

Abiotic stresses, including salt, drought and extreme temperatures, can induce the rapid generation and accumulation of reactive oxygen species (ROS) that cause secondary oxidative stress to plants. Therefore, improving the ROS scavenging capacity is vital for plants to resist abiotic stress conditions. Both POD and SOD are important ROS scavenging enzymes integral to plant stress tolerance. In the present study, two results must be considered together: transgenic *TaeIF5A1* lines had significantly higher POD and SOD activity than WT plants (Figure 9B, C); and transgenic *TaeIF5A1* lines exhibited elevated protein synthesis (Figure 9A) under stress conditions. Consequently, we hypothesize that *TaeIF5A1* increases SOD and POD activity by elevating POD and SOD enzyme synthesis and/or the synthesis of related proteins. In addition, elevated POD and SOD activity may enhance the ROS scavenging capacity of plants under salt stress. Moreover, abiotic stresses in plants also typically result in cell membrane damage, leading to electrolyte leakage. Therefore, electrolyte leakage is a common indicator of membrane damage and leakage is closely related to a loss in water potential. Our results indicate that the level of electrolyte leakage in WT plants is significantly higher than in transgenic plants (Figure 9D). These results suggested that *TaeIF5A1* may also serve a role in membrane protection under stress conditions.

Chlorophyll is the green plant pigment that absorbs light energy vital for photosynthesis. Salinity causes a reduction in chlorophyll levels and inhibits the net photosynthetic rate. Thus, chlorophyll content is a good indicator of the photosynthetic function of plants under adverse environmental conditions. Previous studies have shown that RCC in trees is reduced by aggravated salt stress due to the degradation of enzymatic chlorophyll [28]. We found that the RCC in WT and *TaeIF5A1-*transformed plants was similar under normal conditions. However, *TaeIF5A1*-transformed plants displayed an increased RCC following stress, and RCC was significantly higher in transgenic relative to WT plants following 4–14 days of stress (Figure 9E). These results suggest that eIF5A also has a role in preventing chlorophyll loss under salt stress. The increased RCC indicates that *TaeIF5A1*-transformed plants should increase or maintain a stable photosynthetic rate under salt stress compared to WT plants. A previous study showed that *RceIF5A* confers tolerance to heat, oxidative and osmotic stress, and overexpression of *RceIF5A* can enhance SOD activity and proline level, and decrease electrolyte leakage [16]. In the present study, we further showed that *TaeIF5A1* is also tolerant to salt and heavy metal stresses, and overexpression of *TaeIF5A1* can not only enhance SOD activity and decrease electrolyte leakage, but also facilitate protein synthesis, increase POD activities and maintain higher chlorophyll content under salt stress. These results suggested that *eIF5A* is involved in eliciting a stress response mechanism that may play a common role in plant tolerance to salt, heat, oxidative, osmotic and heavy metal stresses.

## Conclusion

In summary, *TaeIF5A1* is a stress responsive gene that forms part of the ABA signal transduction pathway. The expression of *TaeIF5A1* is likely regulated by the transcription factor TaWRKY and TaRAV both of which can bind to W-box “CTGACT”. Furthermore, *TaeIF5A1* can facilitate protein synthesis and confer abiotic stress tolerance. We propose that *TaeIF5A1* increases plant salt tolerance *via* several physiological pathways, including enhancement of protein synthesis, elevation of SOD and POD activity, increase or maintenance of photosynthetic rates and the protection of cell membranes. Therefore, *eIF5A* serves essential and multiple roles in the reduction and elimination of stress imposed on plants by various abiotic factors.

## Methods

### Plant materials, growth conditions, and treatments

*T. androssowii* seedlings were grown in pots containing a mixture of turf peat and sand (2:1 v/v). Thoroughly watered 2-month-old seedlings were each exposed to the following treatments: 0.4 M NaCl, 20% (w/v) PEG6000, 0.3 M NaHCO_3_, 150 μM CdCl_2_, and 150 μM ABA for 0, 6, 12, 24, 48, and 72 h, respectively. Following these treatments, leaves, stems and roots of seedlings from each sample (sample size of 10 seedlings) were harvested at the indicated times after initiation of each treatment and pooled for real-time RT-PCR analyses.

Seedlings of *Arabidopsis* were grown into pots filled with perlite/soil mixture in a growth chamber under the controlled conditions (16 h light: 8 h dark; 70-75 % relative humidity; 22°C). Three week-old seedlings were each exposed to the following treatments: 20% (w/v) PEG6000, or 100 μM ABA for 0, 3, 6 and 12 h, respectively. After these treatments, the leaves and roots of seedlings were respectively harvested and pooled (sample size of 10 seedlings) for real-time RT-PCR analyses.

### Cloning and expression analysis of *TaeIF5A1*

A *TaeIF5A1* (AY587771) gene was cloned from *T*. *androssowii* cDNA library [29]. The sequence alignments of the eIF5A proteins from different species, including plants, yeast, mammalian and other eukaryotes was conducted using CLUSTALX1.81, and a phylogenetic tree was constructed using the Neighbor-Joining method provided by the computer program MEGA5. The promoter of *TaeIF5A1* was PCR-amplified from genomic DNA of *T. androssowii* using the Genome Walking Kit (TaKaRa, China). To analyze the activity of promoter of *TaeIF5A1*, the 35S promoter in pCAMBIA1301 was replaced with the *TaeIF5A1* promoter (1,486 bp in length) to drive the *β-glucuronidase* (*GUS*) gene (Figure 3A). The *TaeIF5A1* promoter*::GUS* construct was transferred into *Arabidopsis* plants by floral dip method. The T_3_ seedlings were employed for spatial expression analysis of *TaeIF5A1* using GUS staining.

Real-time RT-PCR was performed in Opticon 2 System (Bio-Rad, Hercules, CA) with *α-tubulinβ-tubulin* and *β-actin* genes as internal references. Primers used for RT-PCR are listed in Additional file 3: Table S1. The amplification was performed using the following cycling parameters: 94°C for 30 s followed by 45 cycles at 94°C for 12 s, 60°C for 30 s, 72°C for 40 s and 82°C for 1 s for plate reading. A melting curve was generated for each sample at the end of each run to assess the purity of the amplified products. Each reaction was conducted in triplicate to ensure reproducibility of results. Expression levels were calculated from the cycle threshold according to the delta delta Ct method [30].

### Subcellular localization of the TaeIF5A1 protein

The *TaeIF5A1* coding region without the termination codon was ligated in frame to N-terminal of the green fluorescent protein (GFP) to generate the *TaeIF5A1::GFP* fusion gene. A CaMV 35S promoter was employed to drive *TaeIF5A1::GFP*, and the *GFP* gene under the control of the CaMV 35S promoter (*35S::GFP*) was used as a control. The constructs were introduced into the onion epidermis cells by particle bombardment (Bio-Rad). The transformed cells were analyzed using confocal laser scanning microscopy LSM410 (Zeiss, Jena, Germany).

### Identification of the upstream regulator of *TaeIF5A1*

One W-box motif (“CTGACT”) [21] was found to exist in the promoter of *TaeIF5A1* (Additional file 1). To study which gene can recognize this W-box and regulate the expression of *TaeIF5A1*, the three tandem copies of promoter sequence fragment (“AGGCTGACT”) containing W-box motif sequence were cloned into a pHIS2 vector (construct R3, Figure 5A), and were screened with *Tamarix* cDNA library for a one-hybrid assay (Clontech, Palo Alto, CA, USA). To investigate the interactions between the W-box and positive clones, we mutated the W-box core motif “TGAC” [31,32] with “TGGC”, “TAAC” or “TTTT” (constructs R4, R5, R6, Figure 5A) , and the interactions between the mutant W-box sequences and the positive clones were performed using yeast one hybrid. To further confirm the upstream regulator of *TaeIF5A1*, a 461 bp fragment of *TaeIF5A1* promoter (from −456 to −916) containing the W-box motif (construct R1, Figure 5A ), and a 165 bp fragment of *TaeIF5A1* promoter (from −591 to −755) containing the W-box motif and a 165 bp fragment of *TaeIF5A1* promoter (from −591 to −755) containing the mutated W-box core motif “TTTT” (construct R2, *mR2*, Figure 5A), were cloned into pHIS2, respectively. The interactions between putative upstream regulators and the promoter fragments containing W-box or mutated W-box were performed using a yeast one-hybrid assay. In the above experiments, the p53HIS2 plasmid (pHIS2 contains three copies of p53 DNA element) was used as a negative control vector. All primers are shown in Additional file 3: Table S2.

For further verification of these interactions, the three tandem copies of the W-box and the 165 bp promoter fragments containing W-box motif or mutated core motif “TTTT” were fused with 35S CaMV −46 minimal promoter and respectively cloned into pCAMBIA1301 to replaced with its 35S promoter for driving the *GUS* gene (constructs containing three tandem copies of the W-box named as pCAM-W-box, containing promoter fragment with W-box and mutated W-box named as pCAM-W165 and pCAM-mW165). The effector vectors were constructed by cloning the full ORF of *TaRVA* or *TaWRKY* into pROKII under the control of 35S promoter (named as pROKII-TaRVA and pROKII-TaWRKY) (Figure 5 Da). All primers are shown in Additional file 3: Table S3. Both the reporter and effector vectors were co-transformed into tobacco leaves using the particle bombardment. GUS staining assay was performed as described by Jefferson [33], and GUS activity was determined according to the method of Jefferson [34].

To investigate the expression patterns of the upstream regulators of *TaeIF5A1*, real-time PCR was performed to determine the expression of *TaeIF5A1* and the upstream regulators in *Tamarix* under ABA and osmotic stress conditions. For investigation of the expression of the homologs of *TaeIF5A1*, *TaRAV* and *TaWRKY* in *Arabidopsis* in response to ABA and osmotic stimulus, BLASTX research on Tair ( http://www.arabidopsis.org/Blast/) was performed, we identified the homologs of *TaeIF5A1*, *TaRAV* and *TaWRKY* in *Arabidopsis* are AT1G13950, AT1G68840 and AT1G13960, respectively. An *actin* gene (AT3G18780) was used as internal reference to normalize the amount of total RNA present in each reaction. The primers used are listed in Additional file 3: Table S1, and the real-time PCR conditions were the same as above.

### Expression of *TaeIF5A1* in *S. cerevisiae* and stress-tolerance assays

The full ORF of *TaeIF5A1* was cloned into pYES2 vector (Invitrogen), and was introduced into *S. cerevisiae* strain, INVSc1 (*MATa*, *his3-1*, *leu2*, *trp1-289*, *ura3-52*. His-, Leu-, Trp-, and Ura-). To determine the expression peak of *TaeIF5A1* in yeast, yeast transformants harboring the *TaeIF5A1* were cultivated in induction medium (SC-U medium containing 2% galactose) at 30°C for 0, 12, 24, 36, 48 and 60 h, and harvested for RNA gel blot analysis.

For stress tolerance assays, clones harboring *TaeIF5A1* and empty pYES2 (control) were cultured into SC-U medium containing 2% glucose at 30°C with overnight shaking, adjusted to OD_600_ of 0.4 in induction medium, and incubated at 30°C for 36 h (RNA gel blot result showed that peak level of exogenous gene induced at this time). After incubation, cell densities were adjusted to equal and incubated in different concentrations of NaCl, KCl, LiCl or sorbitol, then they were incubated at 30°C with overnight shaking. The growth rates were evaluated by measuring the OD_600_ for liquid medium in each sample.

To analyze protein content, yeast transformants harboring *Peroxiredoxin* gene (*TaPrx1*, GenBank number: JQ082512) from *Tamarix* were used as control (it can remove the protein synthesis differences between the yeast transformants harboring *TaeIF5A1* and empty pYES2; since transformants harboring empty pYES2 failed in producing an exogenous gene-eIF5A compared with that harboring *TaeIF5A1*). The yeast transformants harboring *TaeIF5A1TaPrx1* and empty pYES2 were cultured in induction medium at 30°C for 0, 12, 24, 36 and 48 h, adjusted to equal quantity, and harvested for soluble protein content analysis. The experiment was repeated at least three times. The protein extraction followed the procedure described by Kushnirov [35] and protein content analyses were performed following the Bradford method [36].

### Construction of plant expression vector and poplar transformation

The *TaeIF5A1* was cloned into pROKII (Additional file 2A), in which *TaeIF5A1* under the control of CaMV 35S promoter, and transferred into poplar plants (*Populus davidiana* Dode *× P. bollena* Lauche) using the *Agrobacterium*-mediated transformation. Kanamycin-resistant lines were detected by DNA gel blot and RNA gel blot. DNA probe for RNA and DNA gel blot were prepared by PCR amplification of the coding region of the *TaeIF5A1* using digoxigenin (DIG) - PCR labeling mix (Roche). Total DNA (30 μg) from samples was digested with *BamH* I and *Sac* I and separated by electrophoresis on a 0.8% agarose gel. The DNA was denatured with NaOH and then transferred to Hybond N^+^ membranes (Amersham). Hybridization and detection was performed following the manual instruction (DIG High Prime DNA Labeling and Detection Starter Kit II; Roche). To detect the expression of exogenous *TaeIF5A1*, total RNA (20 μg) was fractionated on formaldehyde agarose gels, blotted on Hybond N^+^ membranes and fixed by UV cross-linking (254 nm, 8 min). Hybridization and detection were conducted following the manufacturer’s instructions (Dig Northern starter kit, Roche).

### Physiological analysis of transgenic and nontransgenic poplar

The wild-type and the transgenic plants exhibiting similar height (about 1 cm in length) were grown on 1/2MS medium supplemented with 0.6% NaCl (16 h light: 8 h dark, 25°C in tube). The phenotypes of plantlets were photographed, and the heights of plantlets were measured after 20 d of growth.

For growth comparison of plants in soil, plantlets from WT and transgenic plants with similar height (about 70 cm in height) were employed. The height and basal diameter of each sample (sample size of 10) were measured before stress as baseline values. The plantlets were treated with 0.8% NaCl solution for 30 d then watered normally. Following 90 days of growth, the height and basal diameter (final values) were measured, and the relative growth rates of growth in height or basal diameter were calculated.

For physiological analysis, plantlets (60–100 cm in height) from WT and transgenic plants grown in soil were watered with 0.8% (w/v) NaCl solution for 0, 1, 4 and 7 d, and leaves were harvested for analyses. For measurement of concentration of soluble protein, a standard curve for protein level with known concentrations of bovine serum albumin (0–100 mg, at 20 mg intervals) was generated. Phosphate buffer (1.5 mL, 0.01 M, pH 7.0) was added with sample leaf powder (0.1 g), extracted for 3 min, and centrifuged. One mL of supernatant was added with 2 mL of coomassie brilliant blue G250 regent, and the light absorbance was determined at 595 nm. Water was used instead of supernatant as control, and the protein concentrations were calculated using the standard curve. For POD activity measurement, each sample powder (0.05-0.1 g) was incubated with 1.5 ml of 0.01 M phosphate buffer (pH 7.2) at 4°C for 30 min. After centrifugation, 20 μL of supernatant was diluted to 500 μL with water, then added with 0.5 mL of 0.8% H_2_O_2_ , 0.5 mL of 0.1 M phosphate buffer, 0.5 mL of 0.1 M Guaiacol buffer, and incubated at 30°C for 8 min. Light absorbance (ΔA_470_) of the reaction solution was measured at 470 nm. Water was used instead of H_2_O_2_ as a control. POD activity (*A*_*pod*_) was calculated as follows: *A*_*pod*_ = (ΔA_470_*V*) / *WTv* × 100. Where *V*: total enzyme volume, *v*: the volume of enzyme used in reaction, *W*: the material weight, *T:* reaction time (min). For SOD activity assay, phosphate buffer (1.5 mL) was added with the leaf powder and incubated at 4°C for 30 min. After centrifugation, 30 mL of the supernatant was diluted to 500 mL with water and added with 1.5 mL of reaction buffer (0.013 M Met, 6.3 × 10^-6^ M NBT, 6.5 × 10^-6^ M riboflavin, 1 × 10^-4^ M EDTA, 0.05 M phosphate buffer, pH 7.8), and incubated at 30°C for 10 min under 6000 LX. The solution was measured at 560 nm. SOD activity was calculated as A_SOD_[Ug^-1^ min-1 (FW)] = (ΔA560 × N) / (50% WT); where ΔA560 is the decrease absorbance at 560 nm (%), N: the dilution folds, W: the weight and T: the reaction time (min). Electrolyte leakage was determined according to Wang *et al.*[37]. Soluble protein contents were measured following Bradford method [36]. A chlorophyll analyzer (Konica Minolta, Japan) was used to determinate relative chlorophyll content (RCC) in plants stressed for 1–14 d. Each sample contained at least ten plantlets and each experiment was performed in triplicate to ensure the accuracy of analyses.

For other abiotic stress tolerance tests, the plantlets with similar size were grown on 1/2MS medium supplied with 300 μM of CuSO_4_, CdCl_2_, 1 mM of ZnCl_2_ and 200 mM of sorbitol. Plantlets growing in normal 1/2MS medium were used as the control. After 16 d of stress, the height between WT and transgenic lines plants were compared.

### Statistical analysis

Data analyses were carried out using SPSS 16.0 (SPSSInc, Chicago, III, USA) software. For all the analyses, the significance level was set at P < 0.05. Sample variability is given as the standard deviation (S.D.) of the mean.

## Authors’ contributions

YW conceived and designed the experiments. LW, CX and CW performed the experiments. LW, CX and CW analyzed the data. YW contributed reagents/materials/analysis tools. LW and YW wrote the manuscript. All authors read and approved the final manuscript.

## Supplementary Material

Additional file 1 **The promoter sequence of TaeIF5A1 and the cis-elements within the promoter.** The *cis*-elements are shown in different colors and the PCR primers used for the amplification of promoter fragments used in the yeast one-hybrid assay are indicated by a solid line. The primers Pro-af and Pro-ar were used amplifying 461 bp promoter fragment, and Pro-bf and Pro-br were used amplifying 165 bp promoter fragment. The putative transcription start site is underlined and the start codon (ATG) is labeled with a rectangle. Click here for file

Additional file 2 **DNA and RNA gel blot analyses of TaeIF5A1-transformed poplars.** A. Diagram of the T-DNA region of the pROKII-TaeIF5A1 vector used for transformation. B. DNA gel blot analysis of transformed plants. DNA (30 μg) from each sample was digested with *Bam*H I and *Sac* I, separated on agarose gels, denatured and transferred to Hybond N^+^ membranes. C. RNA gel blot analysis of WT and the transgenic poplar plants. Total RNA (20 μg) from each sample was fractionated on formaldehyde agarose gel and blotted on Hybond N^+^ membranes. P, pROKII-TaeIF5A1 vector using as positive control; WT, wild type poplar plants; 1–10, ten lines of transgenic poplar plants. Click here for file

Additional file 3 Primers used in the study. Click here for file
