# Supplementary material for: Characterization of a eukaryotic translation initiation factor 5A homolog from Tamarix androssowii involved in plant abiotic stress tolerance
Source: BMC Plant Biol. 2012 Jul 26;12:118. doi: 10.1186/1471-2229-12-118 (PMC3479025; doi:10.1186/1471-2229-12-118)
Supplement: Additional file 1 — The promoter sequence of TaeIF5A1 and the cis-elements within the promoter. The cis-elements are shown in different colors and the PCR primers used for the amplification of promoter fragments used in the yeast one-hybrid assay are indicated by a solid line. The primers Pro-af and Pro-ar were used amplifying 461 bp promoter fragment, and Pro-bf and Pro-br were used amplifying 165 bp promoter fragment. The putative transcription start site is underlined and the start codon (ATG) is labeled with a rectangle. [file 1471-2229-12-118-S1.doc]

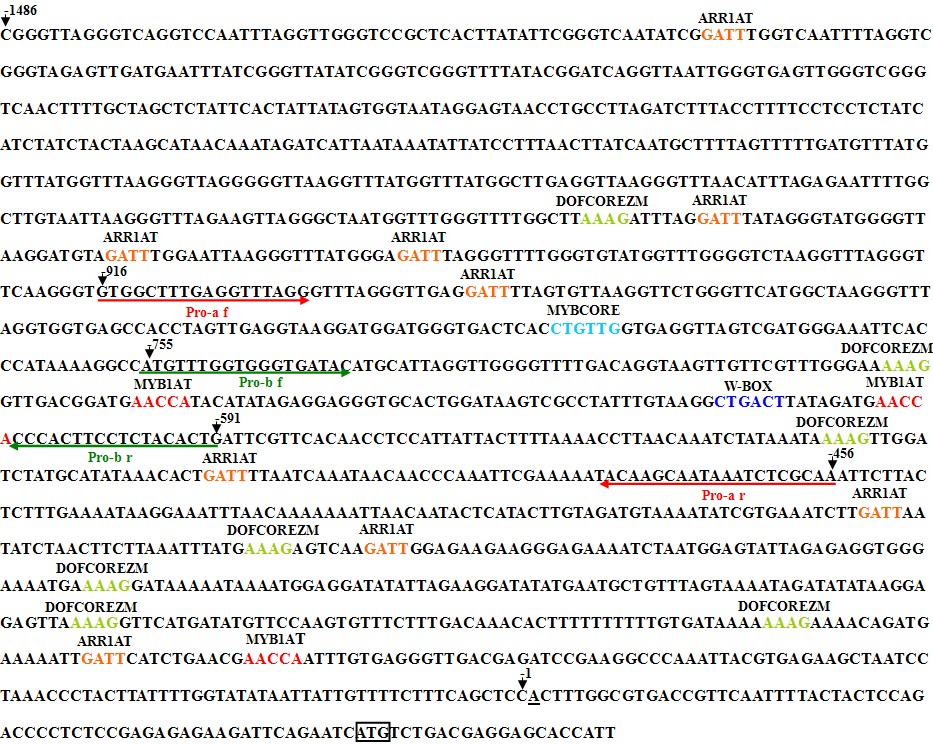


**Additional file 1** – **The promoter sequence of *TaeIF5A1* and the *cis*-elements within the promoter*.***

The *cis*-elements are shown in different colors and the PCR primers used for the amplification of promoter fragments used in the yeast one-hybrid assay are indicated by a solid line. The primers Pro-af and Pro-ar were used amplifying 461 bp promoter fragment, and Pro-bf and Pro-br were used amplifying 165 bp promoter fragment. The putative transcription start site is underlined and the start codon (ATG) is labeled with a rectangle.
