# Supplementary material for: Characterization of a eukaryotic translation initiation factor 5A homolog from Tamarix androssowii involved in plant abiotic stress tolerance
Source: BMC Plant Biol. 2012 Jul 26;12:118. doi: 10.1186/1471-2229-12-118 (PMC3479025; doi:10.1186/1471-2229-12-118)
Supplement: Additional file 2 — DNA and RNA gel blot analyses of TaeIF5A1-transformed poplars. A. Diagram of the T-DNA region of the pROKII-TaeIF5A1 vector used for transformation. B. DNA gel blot analysis of transformed plants. DNA (30 μg) from each sample was digested with BamH I and Sac I, separated on agarose gels, denatured and transferred to Hybond N+ membranes. C. RNA gel blot analysis of WT and the transgenic poplar plants. Total RNA (20 μg) from each sample was fractionated on formaldehyde agarose gel and blotted on Hybond N+ membranes. P, pROKII-TaeIF5A1 vector using as positive control; WT, wild type poplar plants; 1–10, ten lines of transgenic poplar plants. [file 1471-2229-12-118-S2.doc]

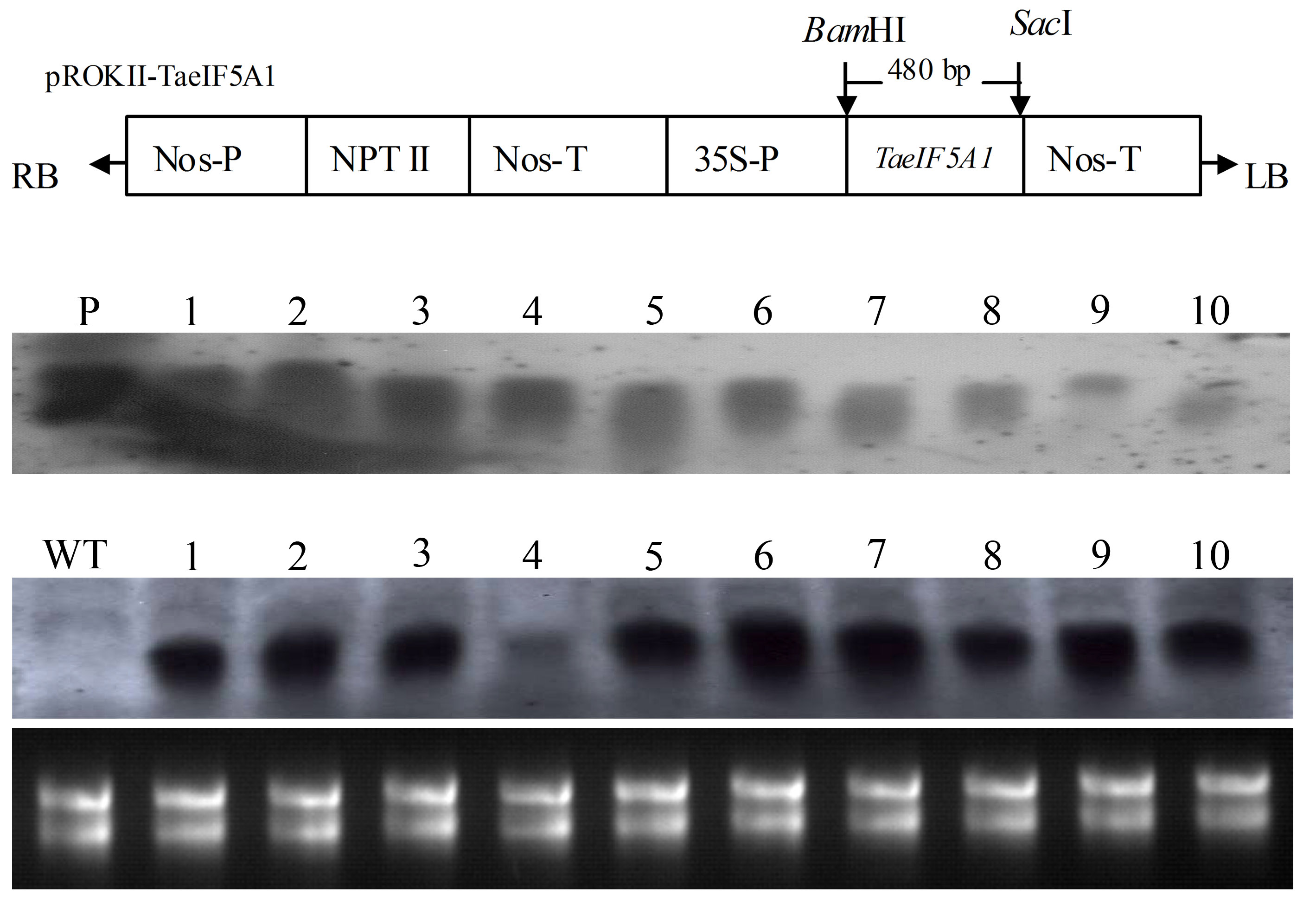


**A**

**B**

**C**

**Additional file 2** – **DNA gel blot and RNA gel blot analysis of *TaeIF5A1*-transformed poplars.**

A. Diagram of the T-DNA region of the pROKII-TaeIF5A1 vector used for transformation. B. DNA gel blot analysis of transformed plants. DNA (30 µg) from each sample was digested with *Bam*H I and *Sac* I, separated on agarose gels, denatured and transferred to Hybond N^+^ membranes for hybridation. C. RNA gel blot analysis of WT and the transgenic poplar plants. Total RNA (20 µg) from each sample was fractionated on formaldehyde agarose gel and blotted on Hybond N^+^ membranes. P, pROKII-TaeIF5A1 vector using as positive control; WT, wild type poplar plants; 1–10, ten lines of transgenic poplar plants.
