# Supplementary material for: Characterization of a eukaryotic translation initiation factor 5A homolog from Tamarix androssowii involved in plant abiotic stress tolerance
Source: BMC Plant Biol. 2012 Jul 26;12:118. doi: 10.1186/1471-2229-12-118 (PMC3479025; doi:10.1186/1471-2229-12-118)
Supplement: Additional file 3 — Primers used in the study. [file 1471-2229-12-118-S3.doc]

**Additional file 3 – Primers used in the study.**

**Table S1** Primers used for real-time RT-PCR.

| Genes | GenBank number | Forward and Reverse Primers (5’-3’) | |
| --- | --- | --- | --- |
| *TaeIF5A1* | AY587771 | cacatcgtcatcaagaaccg | CCACCAGTCTCAGTCAGAAG |
| *β-Actin* | FJ618517 | AAACAATGGCTGATGCTG | ACAATACCGTGCTCAATAGG |
| *α-tubulin* | FJ618518 | CACCCACCGTTGTTCCAG | ACCGTCGTCATCTTCACC |
| *β-tubulin* | FJ618519 | GGAAGCCATAGAAAGACC | CAACAAATGTGGGATGCT |
| *TaRAV* | JQ040809 | AGGACGGGAATGATAAGG | GTCAGCACGTAGCTCTGG |
| *TaWRKY* | JQ040808 | AACAAAGATAGCCAGTAAC | CTGAATAAGCCCTCGTA |
| AT1G13950 | AT1G13950 | GAGGTTTCAACCTCGAAGAC | TCCATCATCAAACCCACTC |
| AT1G68840 | AT1G68840 | GAATCTTTCTCCGCCACC | CTAAATCGCCGTCTTCCA |
| AT1G13960 | AT1G13960 | TGTCTGAGGCAAGTGACGGTG | AGTAGCTCCTCGGATAAGG |
| *Actin* | AT3G18780 | TCTTCTTCCGCTCTTTCTTTCC | TCTTACAATTTCCCGCTCTGC |

**Table S2 Primer sequences employed in the yeast one-hybrid assay.**

| construct | Forward and Reverse Primers (5’-3’) | |
| --- | --- | --- |
| R1 | GGAATTCGTGGCTTTGAGGTTTAGG | CGAGCTCTTGCGAGATTTATTGCTTGT |
| R2 | GGAATTCATGTTTGGTGGGTGATAC | CGAGCTCCAGTGTAGAGGAAGTGGG |
| *mR2* | GGAATTCATGTTTGGTGGGTGATAC | CGAGCTCCAGTGTAGAGGAAGTGGGTGGTTCATCTATAAAAAAGCCTTACAAATAGGCGACTTAT |
| R3 | AATTCAGGCTGACTAGGCTGACTAGGCTGACTAGGGAGCT | CCCTAGTCAGCCTAGTCAGCCTAGTCAGCCTG |
| R4 | AATTCAGGCTGGCTAGGCTGGCTAGGCTGGCTAGGGAGCT | CCCTAGCCAGCCTAGCCAGCCTAGCCAGCCTG |
| R5 | AATTCAGGCTAACTAGGCTAACTAGGCTAACTAGGGAGCT | CCCTAGTTAGCCTAGTTAGCCTAGTTAGCCTG |
| R6 | AATTCAGGCTTTTTAGGCTTTTTAGGCTTTTTAGGGAGCT | CCCTAAAAAGCCTAAAAAGCCTAAAAAGCCTG |

**Table S3** Primers employed in the construction of reporter and effector plasmids.

| construct | Forward and Reverse Primers (5’-3’) | |
| --- | --- | --- |
| pCAM-W-box | AGCTTCTGACTCTGACTCTGACTACCCTTCCTCTATATAAGGAAGTTCATTTCATTTGGAGAGAACACGGC | CATGGCCGTGTTCTCTCCAAATGAAATGAACTTCCTTATATAGAGGAAGGGTAGTCAGAGTCAGAGTCAGA |
| pCAM-W165 | CCCAAGCTTATGTTTGGTGGGTGATAC | CATGCCATGGCCGTGTTCTCTCCAAATGAAATGAACTTCCTTATATAGAGGAAGGGTCAGTGTAGAGGAAGTGGG |
| pCAM-mW165 | CCCAAGCTTATGTTTGGTGGGTGATAC | CATGCCATGGCCGTGTTCTCTCCAAATGAAATGAACTTCCTTATATAGAGGAAGGGTCAGTGTAGAGGAAGTGGGTGGTTCATCTATAAAAAAGCC |
| pROKII-TaRVA | CTCTAGAGGATCCCCAGCGACGTCGGGAAGCTG | TCGAGCTCGGTACCCTCATAGAGCTCCAACCACC |
| pROKII-TaWRKY | CTCTAGAGGATCCCCATGGAGATTAAAGAGGTG | TCGAGCTCGGTACCCTTACTGGCTATCTTTGTTAG |
